# Case report: Scapulohumeral arthrodesis in a reindeer

**DOI:** 10.3389/fvets.2023.1270471

**Published:** 2023-11-27

**Authors:** Kimery L. Hankins, Shannon K. Reed, Keila K. Ida, Jeffrey P. Watkins, Sarah A. White

**Affiliations:** ^1^College of Veterinary Medicine, Texas A&M University, College Station, TX, United States; ^2^Veterinary Clinical Sciences, College of Veterinary Medicine, Texas A&M University, College Station, TX, United States; ^3^Faculty of Veterinary Medicine, Texas A&M University, College Station, TX, United States

**Keywords:** reindeer, scapulohumeral joint, subluxation, arthrodesis, locking compression plate™

## Abstract

This case report describes the anesthetic, surgical, and postoperative management of scapulohumeral arthrodesis in a juvenile reindeer with severe lameness due to a chronic proximal humeral fracture and scapulohumeral luxation. The reindeer was managed with prolonged stall confinement and physical therapy and 9 months postoperatively was walking and bearing weight equally when standing. This case demonstrates that comparative techniques from other veterinary species coupled with considerations for reindeer anesthesia can be successful in restoring functional soundness after scapulohumeral arthrodesis.

## Introduction

Relatively few reports of scapulohumeral arthrodesis exist in large animals, in part due to the technicality of the procedure, lack of implant strength, and significant biomechanical forces on the joint. Severe osteoarthritis and traumatic luxation or subluxation of the joint are the most common reasons for the procedure. Although the overall use of scapulohumeral arthrodesis in large animals is not truly known, it is infrequent (1, 2). Scapulohumeral joint instability in large animals can be difficult to diagnose because of significant musculature within this region. Definitive diagnosis is usually achieved through radiographs or computed tomography. Described stabilization techniques in large animals include closed reduction with or without arthroscopy of the joint, open reduction and internal fixation with tension wires, or arthrodesis of the scapulohumeral joint; also recently, a glenoid ostectomy was successfully described in a miniature donkey (3–6). Complications or failure of reduction techniques are common. Scapulohumeral arthrodesis is often considered a salvage procedure and usually reserved for smaller equids. In small animal practice, scapulohumeral luxation is a common orthopedic problem especially in small canine breeds (5), and there are numerous reports of successful surgical management in dogs (5). There are also reports of such surgery performed on domestic hoofstock such as miniature and small horses, donkeys, and a variety of other farm animals (1–4). The purpose of this report is to describe the surgical technique of a scapulohumeral joint arthrodesis, anesthetic management, and postoperative outcome in a captive reindeer with severe pain associated with chronic humeral fracture and scapulohumeral joint luxation.

## Case presentation

### Clinical history

A 9-month-old, 48.5 kg female intact reindeer was brought to the Texas A&M University Large Animal Hospital with an 8-month history of severe, chronic right forelimb lameness. In addition to the lameness, the reindeer had an acute history of head tremors, bruxism, and an occasional left-sided head tilt which was subsequently diagnosed as antler infection.

### Physical examination

On presentation, the reindeer was Grade 5/5 lame in the right thoracic limb with occasional toe-touching, but no absolute weight-bearing was observed. On palpation, severe atrophy of the infraspinatus and supraspinatus muscles were observed. The shoulder could be manually luxated laterally. The reindeer vocalized, exhibited tremors, and moved away from handlers when the right scapulohumeral joint was manipulated. The left antler was observed to be loose with loss of cover and odor. Occasionally, minor head tremors were observed, not related to stimulation or activity. The reindeer was otherwise bright, alert and responsive, and had an appropriate body condition score (3/5). The animal presented with an implanted microchip with integrated temperature biosensor mechanics which was used throughout hospitalization and anesthesia to monitor body temperature.

### Initial diagnostics

**Figure 1:**
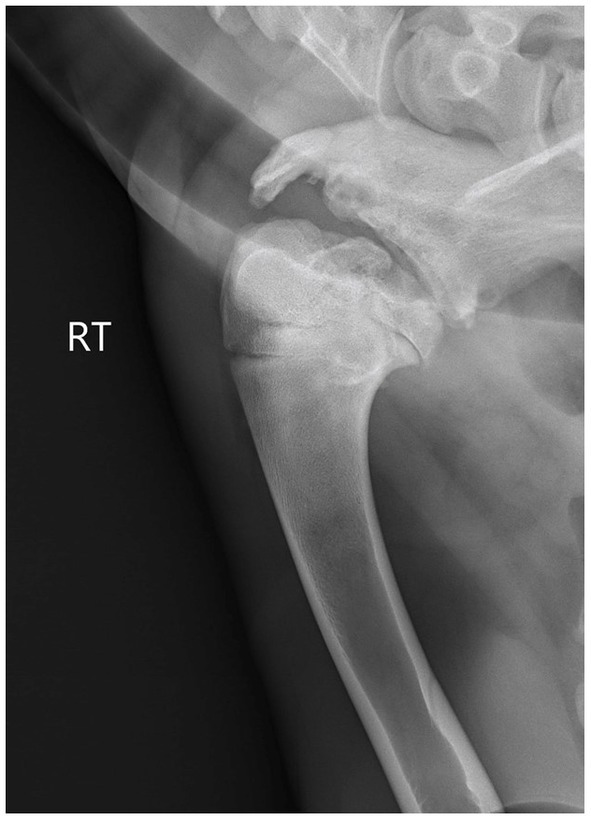
Preoperative radiographs taken on presentation. Note the severe osseous changes to the proximal humeral head and glenoid scapula with significant widening of the joint space.

Radiographs taken at presentation demonstrated the margins of the glenoid of the scapula, head of the humerus, and greater tubercle of the humerus to be undulating and heterogenous with moderate osseous remodeling, sclerosis, and multifocal regions of lucency (Figure 1). The cranial and lateral aspects of the joint were widened significantly. A diagnosis of marked chronic osseous remodeling, osteoarthritis, and instability of the right scapulohumeral joint secondary to proximal humeral fracture was made. Given the history of head tremors and suspected antler infection, skull radiographs were also performed which demonstrated no abnormal findings. Thoracic radiographs were normal. Cerebrospinal fluid analysis via lumbosacral space centesis was performed and was normal. Initial complete blood count and serum biochemistry were unremarkable.

### Anesthesia

Prior to anesthesia and surgery, the patient was treated for the infected antler with ceftiofur crystalline free (subcutaneously once at 6.6 mg/kg) and gentamicin (6.6 mg/kg intramuscularly every 24 h). Poor patient compliance necessitated intravenous catheter placement and the patient was switched to ceftiofur sodium (2.2 mg/kg intravenously every 12 h) and gentamicin (6.6 mg/kg IV every 24 h). The head tremors and discomfort associated with the antler resolved after 5 days and the reindeer was anesthetized for surgical arthrodesis of the right scapulohumeral joint 7 days after presentation. The patient was classified as ASA 2 according to the American Society of Anesthesiologists. Food was withheld for 24 h and water for 10 h, respectively, prior to anesthesia. A 14 gauge over the wire intravenous catheter was placed in the right jugular vein.

Premedication included midazolam at 0.3 mg/kg administered intravenously (IV), which resulted in mild sedation. After flow-by oxygen was provided, the patient was induced with ketamine (2 mg/kg) and propofol (3.5 mg/kg) IV. The tip of a 350-mm straight blade laryngoscope was used to allow visualization of the larynx. The larynx was splashed with 1 mL of 2% lidocaine prior to intubation with a 9 mm internal diameter cuffed endotracheal tube. Cardiovascular and respiratory monitoring was performed with a multiparametric monitor, and parameters were recorded every 5 min. Oxygen saturation using pulse oximetry, continuous electrocardiography, body temperature, invasive arterial blood pressure, end-tidal carbon dioxide (EtCO_2_) using side-stream capnography, and arterial blood gas analysis were monitored. Anesthesia was maintained with sevoflurane (1.7–2.6% expired concentration) in 100% oxygen delivered via a circle system. Mechanical intermittent positive pressure ventilation was instituted soon after intubation to maintain normocapnia (EtCO_2_ 35–45 mmHg) with a tidal volume of 9 mL/kg and a peak inspiratory pressure of 14 cmH_2_O. Lactated Ringers’ solution was administered at a constant rate infusion (CRI) of 5 mL/kg/h intraoperatively. Cooling therapy consisted of an ice bath and ice packs surrounding the patient and under the surgical drapes to minimize intraoperative hyperthermia. Throughout the procedure, body temperature was monitored every 5 min and the ice was removed from around the animal once the temperature fell to 99°F. Intraoperative multimodal analgesia was provided with fentanyl (loading dose of 3 mcg/kg followed by a CRI of 3 mcg/kg/h), ketamine (CRI of 0.5 mg/kg/h), and lidocaine (2 mg/kg loading dose followed by a CRI of 2 mg/kg/h).

Total anesthetic time was 5 h and 30 min with surgical time being 3 h and 45 min. The patient recovered uneventfully.

### Surgical procedure

The patient was induced under general anesthesia and positioned in left lateral recumbency on a bed of ice and padded operating table. The right shoulder region was clipped from the neck to the fifth intercostal space and from the withers to the mid-radius. The clipped area was aseptically prepared with povidone/iodine scrub and isopropyl alcohol. The patient was transported into the operating room and the region was draped aseptically. Prior to the start of surgery, the patient was administered ceftiofur sodium (2.2 mg/kg IV) and gentamicin (6.6 mg/kg IV).

A curvilinear incision using a #10 blade was made through the skin and the subcutaneous tissues extending from the cranial border of the spine of mid-scapula to the mid-humerus. The muscle belly of the brachiocephalicus was incised to reveal the insertion of the biceps muscle. The biceps tendon was transected distal to the supraglenoid tubercle and reflected proximally to reveal the shoulder joint. The supraspinatus muscle was retracted and bluntly dissected away from the cranial margin of the scapular spine to reveal the suprascapular nerve. The suprascapular nerve was isolated and protected using a probe during the placement of the plate. The joint capsule was incised, and the intermediate tubercle was removed using a sharp chisel. Visible and palpable cartilage of the distal scapula and proximal humerus was removed with a curette. A 3.5 narrow, 12-hole, locking compression plate ™ was contoured to the cranial surface of the scapula and the craniolateral surface of the humerus. The plate was secured with four 3.5 mm locking screws, four 3.5 mm cortical screws, and one 4.5 mm cancellous screw (to replace a cortical screw that was stripped during placement). Two of the cortical screws were placed transarticular in lag fashion. The two holes directly centered over the joint and the most distal hole of the plate were left empty. Methyl methacrylate beads infused with florfenicol were applied over the plate.

The transected muscles were closed using 2–0 and 0 polydiaxone (PDS) in a combination of continuous and interrupted patterns. The fascia was closed in a separate layer using 2–0 PDS in a continuous pattern. The subcutaneous tissues were closed using 2–0 PDS in a continuous pattern. The skin was closed using stainless steel skin staples. A tie over bandage was put in place using a laparotomy sponge and secured into place using 0 polypropylene suture rings with umbilical tape laced through the rings over the bandage. The tie over bandage was covered with an iodine impregnated adhesive bandage. A 100 mcg/h fentanyl patch was applied caudal to the right scapula and covered with elastic adhesive tape stapled to the skin. The patient was moved back to her stall where she recovered uneventfully.

### Post-operative care

Postoperative analgesia was administered by placing a fentanyl transdermal patch (100 mcg/h) 7 h after the pre-anesthetic medications were administered. Inflammation was managed postoperatively with flunixin meglumine (2.2 mg/kg IV every 24 h) for 5 days and transitioned to oral meloxicam (generic, 0.5 mg/kg orally every 24 h) for an additional 6 days. Pantoprazole (1 mg/kg IV every 12 h) was administered for 5 days. Ceftiofur sodium (2.2 mg/kg IV every 12 h) was continued postoperatively for 10 days. Gentamicin (6.6 mg/kg IV every 24 h) was discontinued 2 days postoperatively as there were florfenicol impregnated beads at the implant site. At 10 days postoperatively, ceftiofur sodium was discontinued due to thrombophlebitis at the intravenous catheter site. Tulathromycin (2.5 mg/kg subcutaneously once and then again 4 days later) was then given to prevent implant infection.

During recovery and throughout the hospital stay, the reindeer was maintained in a 13 × 13 ft. stall bedded with thick shavings covering a large, gripped mat. During recovery, the reindeer bore weight on her right thoracic limb and was able to ambulate. The reindeer appeared to be doing well and did not show obvious signs of pain or discomfort. The incision remained covered using a modified tie-over bandage for 7 days postoperatively to prevent contamination from the environment. Radiographs of the right scapulohumeral joint obtained 20 days postoperatively demonstrated expected narrowing of the joint, appropriately contoured plate, and no appreciable change in position or breakage of any implants. The reindeer was discharged 21 days postoperatively. Meloxicam (generic, 0.5 mg/kg orally, every 24–48 h) was administered as needed, with the owner reporting no doses given after 4 weeks post-repair.

Initial recommendations stated to keep the patient in a small stall with rubber mats under shavings when not monitored to minimize the risk of slipping. The reindeer was also to be separated from other animals and herd mates during this time. Physical rehabilitation was started during the first month postoperatively by having the reindeer make intentional steps while being led on a halter. Cavaletti poles were set up to provide structured movements and exercise during the recovery period. Approximately 8 weeks postoperatively, the reindeer was noted by the owner to bear weight normally at a walk with minimal lameness, so restriction was discontinued despite recommendations. The owner reported the animal was quite active while turned out with other reindeer, occasionally running about the property.

**Figure 2:**
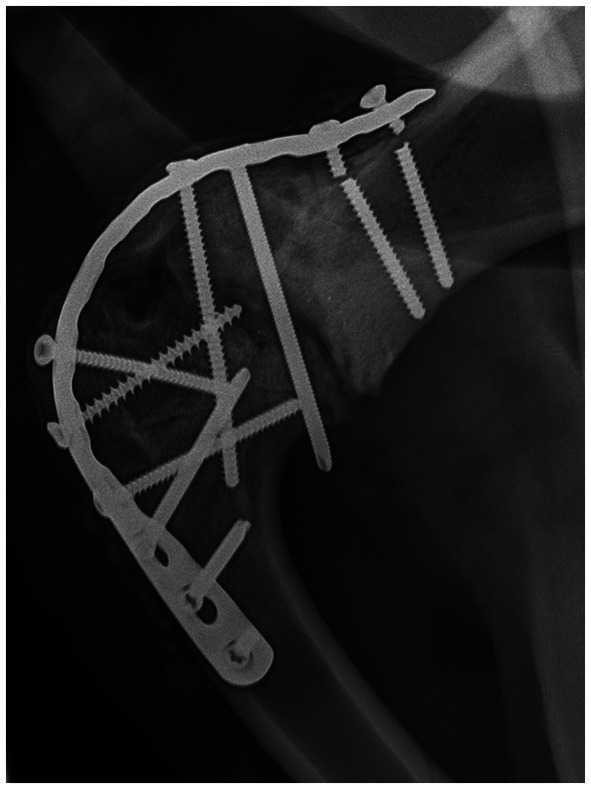
Twelve week recheck right scapulohumeral arthrodesis. The two broken proximal screws are evident. Note the corrected width of the joint space.

At recheck examination approximately 12 weeks after surgery, the reindeer’s vital parameters were within normal limits. The reindeer would bear weight on the affected limb more than 75% of the time. When walking, the reindeer used the limb well without readily apparent lameness, though slight circumduction of the entire limb with each stride was noted. When the reindeer trotted, there were occasional steps of exaggerated circumduction with decreased weight bearing. There was no mobility to the scapulohumeral joint in any direction and no evidence of pain on palpation or manipulation. Radiographs indicated the two most proximal screws were broken at the screw heads, but the degree of shoulder joint fixation was unchanged and adequate (Figure 2). The owner was instructed to return the animal to confinement and perform physical therapy as previously recommended to avoid continued breakdown of the repair.

**Figure 3:**
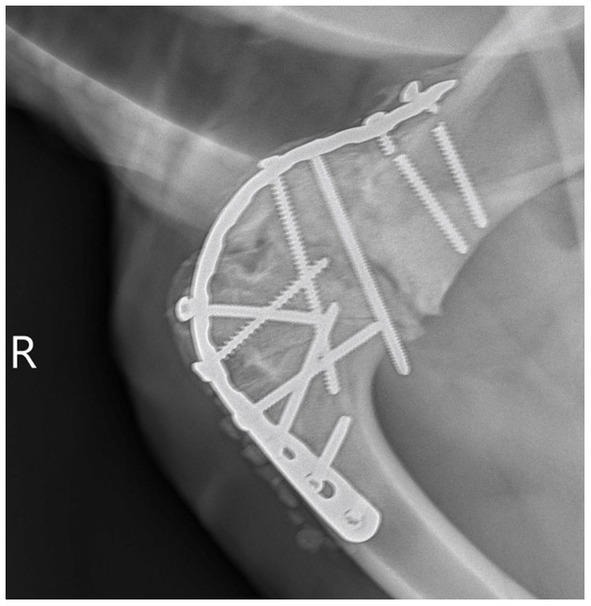
Seventeen week recheck right scapulohumeral arthrodesis. Static progression with no evidence of implant migration. The previously observed broken proximal screws can be visualized.

The animal returned 17 weeks postoperatively and was comfortable using the leg with similar movements to the previous visit. Radiographs showed adequate surgical arthrodesis of the right shoulder with previously described broken screws and no visible migration of the implants (Figure 3). The owner was instructed to gradually increase the turnout time and confinement space allowed.

At 9 months post-surgery, the reindeer was admitted to the hospital for an unrelated medical condition. At that visit, the right scapulohumeral arthrodesis was stable with no pain on palpation, near constant weight bearing, and no discomfort when walking or trotting. The owner was happy with the outcome of surgery and felt the reindeer had a very good quality of life.

## Discussion

This report presents the diagnosis, anesthetic management, surgical treatment, postoperative care, and recheck status of a reindeer presenting to the Food Animal Medicine and Surgery Service at Texas A&M University Veterinary Medical Teaching Hospital in which a scapulohumeral arthrodesis was performed. This report demonstrates a positive outcome based on the restoration of normal ambulation and comfort.

Luxation is more common than primary osteoarthritis within the scapulohumeral joint in miniature horses (3) and can be surgically corrected with a procedure similar to that used in this case. Typically, a favorable prognosis results after joint reduction and can lead to adequate return to function. Although reindeer are not commonly presented for orthopedic surgery, their scapulohumeral joint anatomy is comparable to other hoofstock. The choice between open and closed reduction is made based upon chronicity of lameness, animal size, stability of the joint, and injury complexity. Given this reindeer’s age, size, and chronic injury, open reduction was performed with plate fixation following similar techniques for canine patients (5). Complications of this corrective procedure can include implant failure, chronic lameness secondary to osteoarthritis, limb neuropathy from injury to the suprascapular nerve, and any anesthetic complications specific to ruminants (1).

As noted, scapulohumeral luxations are not unique to a particular species. Usually, joint reduction results in a favorable prognosis for companion, lightweight equine, and other small to medium sized hoofstock. Techniques for arthrodesis of this joint are typically similar across species with minor adjustments made as needed for each animal intraoperatively (1, 3–5, 7). The likelihood of successful outcome depends on factors like the chronicity and complexity of the injury. In most cases, animals will bear weight on the limb within a few months post-surgery (1, 3, 5). The previously described surgical technique of scapulohumeral arthrodesis, made with small adjustments based on the author’s (SR) experience with reindeer, supports this approach for correcting shoulder luxation in other reindeer.

Primary postoperative analgesia for this patient consisted of fentanyl and flunixin meglumine, which was later transitioned to oral meloxicam. Non-steroidal anti-inflammatory drugs (NSAIDs) are recommended after orthopedic procedures as part of a multimodal approach to pain control and as an immediate postoperative inflammatory mediator. However, administration of NSAIDs and the concurrent stress of hospitalization can result in adverse effects such as gastric ulceration. Therefore, a gastroprotectant, such as pantoprazole, is recommended in most postoperative care plans. There is no published documentation of fentanyl patches being used for analgesia in reindeer. Adverse effects published on calves with fentanyl patches include tachycardia, hyperthermia, excitement, and ataxia (8). In sheep (9, 10) and goats (11), the use of a fentanyl patch has not been associated with the development of adverse effects. A transient increase in rectal temperature was not considered clinically significant in goats (12). No adverse effects from the fentanyl patch were noted in this case, and no particular preparation of the skin was required for application other than covering the patch with elastic adhesive tape for security (13–15).

Previous reports of scapulohumeral luxation have described limited rehabilitation protocols, with confinement most commonly recommended. Although objective descriptions of the benefits of specific postoperative techniques do not appear to exist, confinement may be the sole feasible option and is sufficient to ensure a positive outcome. In the current case, specific rehabilitation methods such as stride lengthening exercises using cavaletti poles, lead walking, and weight-shifting exercises likely facilitated the reindeer’s return to function.

Currently, there is no regulation in the United States regarding reindeer entering the food chain for human consumption. While uncommon, there are instances of reindeer being consumed. Therefore, clinicians should formulate therapeutic plans for exotic hoofstock species with extra-label drug use in mind and recognize that animals with implant devices cannot enter the commercial food chain. To communicate this message to clients, withdrawal times for medications administered should be listed in discharge instructions to provide awareness and education on the potential uses of the animal regardless of the client’s intent. Because metal implants, florfenicol infused beads, and gentamicin were used in this animal, the owner was informed that this animal should never enter the food chain.

Case reports offer valuable insights in veterinary literature by providing specific information about procedures that can occasionally be translatable across species. However, it is important to consider their limited statistical significance. Conclusions must be interpreted with the awareness that larger sample sizes will be needed and that not every case is representative of an entire group. Additional studies and comparisons can help clarify whether other techniques for scapulohumeral stabilization following luxation may be suitable for reindeer and provide information for other hoofstock species.

## Conclusion

This report demonstrates that surgical correction via arthrodesis can successfully be used to repair chronic luxation of the scapulohumeral joint in reindeer. Physical rehabilitation therapy is thought to improve mobility and overall outcome. Although there are few reports of successful repair of scapulohumeral arthrodesis in non-traditional species, surgeons should consider applying modifications of techniques published for more traditional species to provide a successful outcome.

## Data availability statement

The original contributions presented in the study are included in the article/Supplementary material, further inquiries can be directed to the corresponding author.

## Ethics statement

All investigations and treatments were performed with the consent of the owners. The nature of this case report did not require ethical approval. The authors confirm compliance with the journal’s ethical guidelines.

## Author contributions

KH: Writing – original draft, Writing – review & editing. SR: Writing – original draft, Writing – review & editing. KI: Writing – review & editing. JW: Writing – review & editing. SW: Writing – review & editing.
