# Supplementary figures and images for: Case report: Scapulohumeral arthrodesis in a reindeer
Source: Front Vet Sci. 2023 Nov 27;10:1270471. doi: 10.3389/fvets.2023.1270471 (PMC10711268; doi:10.3389/fvets.2023.1270471)

# CASE PROGRESSION

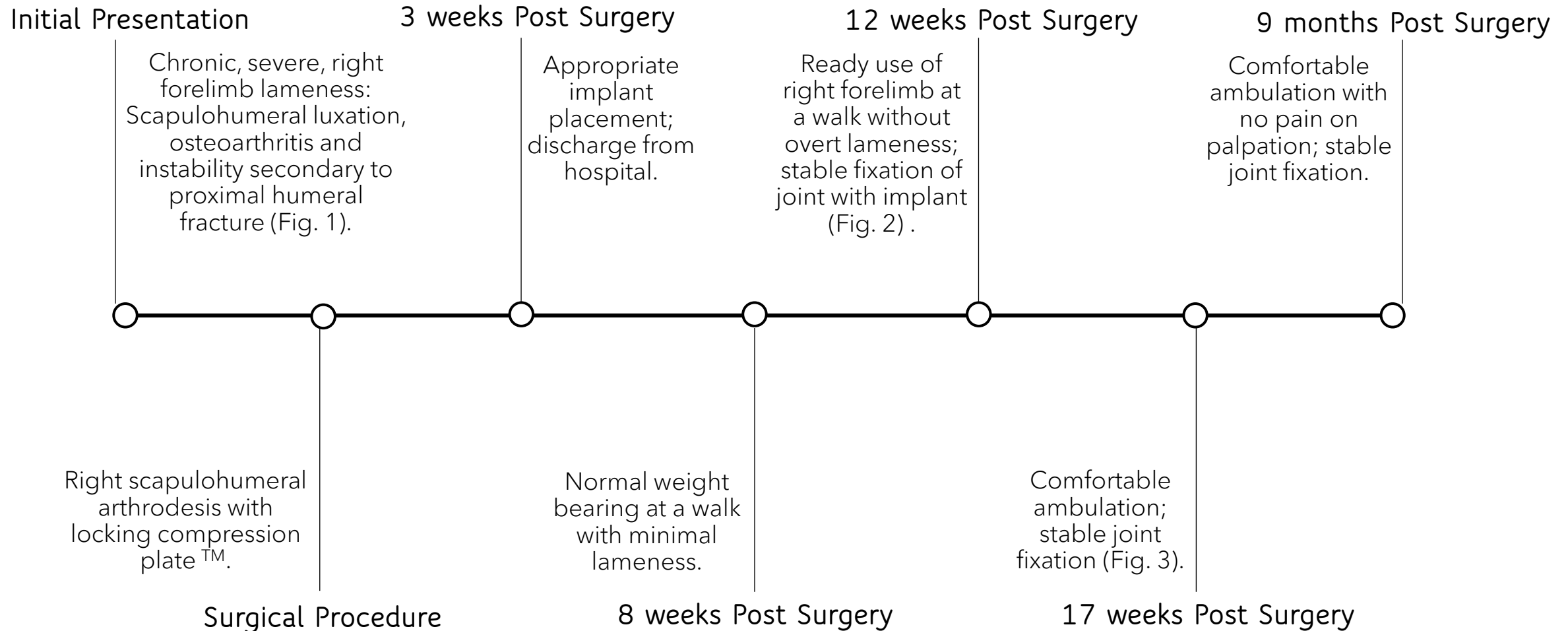

Supplement: Supplementary file 2 [file Data_Sheet_2.pdf]
